# Psychometric evaluation and predictive validity of Ryff's psychological well-being items in a UK birth cohort sample of women

**DOI:** 10.1186/1477-7525-4-76

**Published:** 2006-10-04

**Authors:** Rosemary A Abbott, George B Ploubidis, Felicia A Huppert, Diana Kuh, Michael EJ Wadsworth, Tim J Croudace

**Affiliations:** 1 Department of Psychiatry, University of Cambridge, Box 189, Addenbrooke's Hospital, Hills Road, Cambridge, CB2 2QQ, UK; 2 MRC National Survey of Health and Development, Royal Free & University College Medical School, Department of Epidemiology and Public Health, 1-19 Torrington Place, London, WC1E 6BT, UK

## Abstract

**Background:**

Investigations of the structure of psychological well-being items are useful for advancing knowledge of what dimensions define psychological well-being in practice. Ryff has proposed a multidimensional model of psychological well-being and her questionnaire items are widely used but their latent structure and factorial validity remains contentious.

**Methods:**

We applied latent variable models for factor analysis of ordinal/categorical data to a 42-item version of Ryff's psychological well-being scales administered to women aged 52 in a UK birth cohort study (n = 1,179). Construct (predictive) validity was examined against a measure of mental health recorded one year later.

**Results:**

Inter-factor correlations among four of the first-order psychological well-being constructs were sufficiently high (> 0.80) to warrant a parsimonious representation as a second-order general well-being dimension. Method factors for questions reflecting positive and negative item content, orthogonal to the construct factors and assumed independent of each other, improved model fit by removing nuisance variance. Predictive validity correlations between psychological well-being and a multidimensional measure of psychological distress were dominated by the contribution of environmental mastery, in keeping with earlier findings from cross-sectional studies that have correlated well-being and severity of depression.

**Conclusion:**

Our preferred model included a single second-order factor, loaded by four of the six first-order factors, two method factors, and two more distinct first-order factors. Psychological well-being is negatively associated with dimensions of mental health. Further investigation of precision of measurement across the health continuum is required.

## Background

Recent years have seen a widening interest in research on aspects of well-being [1-4]. Extensive research on subjective well-being (SWB) which focuses mainly on how people feel, e.g. positive affect, negative affect and life satisfaction (see review by Diener et al.) [5], has begun to be complemented by a heightened interest in how well people perceive aspects of their functioning, e.g. the extent to which they feel they are in control of their lives, feel that what they do is meaningful and worthwhile, and have good relationships with others e.g. [6,7]. This perspective is often referred to as psychological well-being (PWB) and is based on a eudaimonic perspective, rather than the hedonic perspective of subjective well-being research.

This new focus has necessitated the theoretical development of new constructs as well as questionnaire items to measure psychological well-being in clinical and population samples. The work of Ryff and colleagues has been at the forefront of this endeavour.

Ryff's scales of Psychological Well-being [8,9] were designed to measure six theoretically motivated constructs of psychological well-being: autonomy – independence and self-determination; environmental mastery – the ability to manage one’s life; personal growth – being open to new experiences; positive relations with others– having satisfying high quality relationships; purpose in life – believing that one’s life is meaningful; and self-acceptance – a positive attitude towards oneself and one’s past life.

Despite the widespread interest in Ryff's theoretical framework, and application of the Ryff PWB items, the psychometric properties of the proposed sub-scales remain contentious. In particular there has been concern over issues of factorial validity and distinctiveness. Do the items intended to measure each theoretical domain, really do so? Do the items capture information from more than one domain? Are fewer dimensions actually revealed by empirical data collected to test the multidimensional theory?

Previous psychometric studies of the Ryff PWB are summarised in Table 1. To date no independent investigation of the factorial validity of Ryff's well-being items has unequivocally supported the *a priori *six-factor structure. Authors of existing studies either challenge the value of so many theoretical constructs, whose scores correlate >0.8 or 0.9, or have not confirmed the fit of the proposed model [10-14]

**Table 1:** Summary of psychometric studies of Ryff's Scales of Psychological Well-being

Author	Date	No. of items	Sample size	Sample	Analysis method	Summary of results
Ryff, CD.	1989	120	321	Community volunteers; 3 age groups: young adults (*mean age = 19.3 years, s.d. 1.6*; midlife (*49.9, 9.4*) and older adults (*75.0; 7.1*). F = 60%.	Correlational analysis	Inter-factor correlations of *a priori *6F model .32–.76; (highest: E with S = .76; S with P = .72; P with G = .72; P with E .66); internal consistency coefficients 0.86–0.93.
Ryff, CD & Keyes, CL.	1995	18	1,108	Midlife in the United States (MIDUS). (*Mean age = 45.6, 14.8*); F = 59%.	LISREL 7.2 PRELIS WLS estimation continuous	Tested 6F model (BIC -167.6 df = 120), 6F with 2^nd ^order (BIC -166.0, df = 129) and unidimensional model (-38.2, df = 135). Inter-factor correlations 0.30–0.85. (Highest E with S .85)
Clark, PJ, Marshall VW, Ryff CD, Wheaton, B.	2001	18	4,960	Canadian Study of Health & Aging (*mean age = 76*)	EQS ML estimation (continuous)	Tested 1-6F models. Found low internal consistency & reliability for all models, some low factor loadings and a large number of cross-loadings. *A priori *6F model (CFI = 0.77). Preferred solution – 6F model with 4 cross-loadings
Kafka GJ & Kozma A.	2002	120	277	Canadian students (*mean age = 21.3, 3.8*) F = 67%.	Principal components analysis with varimax rotation.	Unrestricted model extracted 15 factors; a model restricted to 6F did not correspond to a-priori Ryff dimensions*.
Van Dierendonck, D.	2004	84 54 18 #	233 420	Dutch students (*mean age = 22, 6*); F = 67% Dutch professionals (*mean age *= 36, 8); F = 31%.	CFA – LISREL 8.5 ML estimation	*A-priori *6F with 2^nd ^order returned lower AIC than other models (e.g. 1F, 2F negative and positive, 5F & 6F) but CFI unacceptably low = 0.65 (84-item), 0.73 (54-item); 0.88 (18-item). Factorial validity only acceptable for 18-item version, but internal consistency was below acceptable limits for 84, 54 & 18-item.
		54	420	Dutch professionals (*mean age *= 36, 8); F = 31%.		
Cheng, ST, Chan, AC.	2005	24	1,259	Chinese aged 18–86 (*mean age 44.7, 16.6*); F = 82%, volunteers at hospitals in Hong Kong	CFA – LISREL (8.52)/PRELIS with ML estimation.	6F model (CFI = 0.93); 1F (CFI = 0.88) and 6F + 2^nd ^order (CFI 0.92). Initial CFA on 18-item scale with poorer model fit and low internal consistency than their revised 24-item version.
Springer, KW, Hauser, RM.	2006	42 12 18 18	6,282 6,038 2,731 9,240	Wisconsin Longitudinal Survey MIDUS (25–74 yrs) National Survey of Families and Households NSFH II	CFA – LISREL/PRELIS Estimator = WLS (Polychoric correlations) Adjustment for correlated negative method factor and adjacent questions	Tested a range of models (including 1F, 6F + 2^nd ^order). 6F *a-priori *model with correlated negative method factor, correlated errors for adjacent questions and 3 error correlations had lowest BIC; although model fit criteria was poor. High inter-factor correlations for all dimensions (.72–.97) Highest E with S; P with S.
		18	2,731	MIDUS (25–74 yrs)		
		18	9,240	National Survey of Families and Households NSFH II		
Abbott, RA, Ploubdis, GB, Huppert, FA, Kuh, D, Wadsworth, MJ, Croudace, TJ.	2006	42	1,179	National Survey of Health & Development (UK) Women age 52.	CFA Mplus (3.1) rWLS estimator. Negative & positive method factors (uncorrelated)	Tested a range of models (including random allocation unidimensional, 6F + 2^nd ^order). Method factors (orthogonal to the constructs and each other) for questions reflecting positive and negative item content improved model fit. Preferred model included a single 2^nd ^order factor, loaded by 4 first-order factors (E,G,P,S), two method factors, and 2 distinct first-order factors (A,R)

Sample characteristics are restricted to that derived from original sources. 6F = six-factor model; M = Males; F = females; Age = (*mean, standard deviation*) where known. A = autonomy, E = environmental mastery, G = personal growth, P = purpose in life, R = positive relations with others, S = self acceptance. CFA = confirmatory factor analyses; WLS = weighted least squares; ML = maximum likelihood estimation, rWLS = robust weighted least squares, AIC = Akaike information criteria; CFI = comparative fit index. # In Van Dierendoncks (2004) study the 18-item scale was tested by extracting the relevant questions from the 54-item version.* Kafka & Kozma (2002) – An additional analysis included the Satisfaction with Life Scale (SWLS) and Memorial University of Newfoundland Scale of Happiness (MUNSH). Three factors were extracted with eigenvalues over 1.0. Factor 1 – E,G,P,S; factor 2 – MUNSH & SWLS; factor 3 – A, R.

Many of these studies have reached similar conclusions despite the analysis of different short and long forms of the Ryff scales. As shown in Table 1, versions with different numbers of items have been applied in a variety of settings and samples. The original instrument included 120 items (20 per dimension) but shorter versions comprising 84 items (14 per dimension), 54 items (9 per dimension), 42 items (7 per dimension) and 18 items (3 per dimension) are now widely used. It is important to note that the overlap among items in the alternative versions of the Ryff scales is limited; for example, the 18-item version has only six items in common with the 42-item version, one item for each dimension.

Ryff's own studies [7,9] have reported high correlations among scores for the constructs that were proposed as independent. It is possible that the measures may not, in practice, adequately operationalise the constructs proposed by her theory. For example, in Ryff's first study, which employed 120 items, the inter-correlations among factor scores for the six dimensions ranged from 0.32 to 0.76. Associations were particularly strong between personal growth and purpose in life; self-acceptance and purpose in life; and environmental mastery and self-acceptance [9]. Indeed, the magnitude of these inter-factor associations prompted Ryff and Keyes [7] to estimate a second-order factor model which invoked a general PWB factor to explain associations among the first-order constructs, so clearly they acknowledged the high inter-dependencies among the six factors.

In a psychometric investigation of multi-samples, Springer & Hauser [14] factor analysed Ryff PWB items from three large North American studies; the Wisconsin Longitudinal Survey (42-items and 12-items); MIDUS – Midlife in the United States (18-items) and the National Survey of Families and Households (NSFH II) (18-items). Their results, based on internal construct validity arguments alone, seem to provide yet further evidence that the Ryff PWB items may either measure less than six distinct constructs, or that the theoretical constructs exist at two levels of definition.

Psychometric studies of multi-item questionnaires often see a need to isolate components of response tendency that are due to methodological features e.g. design or wording of items [15,16]. Springer & Hauser [14] introduced a single latent variable (a method factor) to isolate the covariance among responses common to all negatively worded Ryff items. In their study, this component of their model was found to considerably improve model fit. In their response 17 to a commentary on their conclusions by Ryff and Singer 18  they reported a test of a 4-factor model based on the four most highly correlated dimensions (environmental mastery, personal growth, purpose in life and self-acceptance) and compared this to a 4-factor model using the same items but where item allocation was based on positive and negative wording and position (i.e. earlier or later) in the instrument. They demonstrated similar indices of model fit between the two models.

The penultimate column of Table 1 reports the factor analysis method used by existing psychometric studies. Most existing work has examined the dimensionality of Ryff PWB items using the traditional linear factor model, which assumes that responses are continuous scores on an interval scale metric [7,11,12]. Hauser and Springer's analysis [14,17] was performed using a factor model that provide an ordinal/graded treatment of the Likert style response scales. Model estimation was based on polychoric correlation among items and weighted least squares methodologies (WLS). They argued that application of the standard linear model was inappropriate. Application of linear statistical models to ordinal data can result in biased estimates of factor loadings [19-22]. Categorical data factor analyses models are considered to be more theoretically appropriate in their statistical underpinnings for Likert scaled (ordinal) data [23-26]

In addition to these considerations that have focused entirely on issues of internal construct and factorial validity of the Ryff PWB items, it is important to consider evidence for the construct validity of the PWB in relation to other dimensions of mental health and well-being.

Ryff [7] reported correlations from three cross-sectional studies that included measures of happiness, life satisfaction and depression in addition to PWB items. Positive associations were found between measures of happiness and life satisfaction and all PWB dimensions but with the strongest correlations for self-acceptance and environmental mastery. Conversely, the severity of depressive symptoms were negatively associated with all PWB dimensions, but with the strongest negative correlations again evident for environmental mastery and self-acceptance. In a small European sample of Swedish white collar workers (N = 91) Lindfors [27] reported a correlation of -0.61 between the score on a short screening measure for minor psychiatric morbidity (the 12-item General Health Questionnaire) [28] using a total (sum) score from the 18-item Ryff. These results suggest 1) some overlap between reported psychological well-being and the absence of depressive symptoms, and 2) positive associations with other measures of subjective well-being. More external construct validity evidence is desirable since the convergent and divergent validity of PWB measures is still not well-understood. Longitudinal studies of PWB and related constructs are of value since it is of intrinsic interest to examine the consequences of PWB for other outcomes, and to contribute new data on predictive validity, which is currently absent. The existing studies are limited by being based almost solely on concurrent self-report data.

Motivated by the controversy over the dimensionality of Ryff PWB items and methodological developments described in existing studies (Table 1) we aimed to provide the first independent examination of the *a priori *structure of the Ryff PWB items in a UK population-based sample. In doing so we use methods that are theoretically appropriate for factor analysis of ordinal data and compare the fit of models with the following components:

a) single (unidimensional) versus multi-factor (multidimensional) models,

b) incorporation of method factors

c) consideration of hierarchical models with second-order factors

Because few studies have reported any prospective consequence or correlates of population variations in levels of PWB we also examine the predictive validity i.e. the longitudinal association between the PWB constructs and a summary measure of psychological distress comprising the 28-item General Health Questionnaire [29].

## Methods

### Sample

The sample comprised participants from the Medical Research Council's National Survey of Health and Development (NSHD), the 1946 British birth cohort study. The NSHD is a stratified sample of singleton births occurring to married parents in England, Scotland and Wales during the week of 3–9 March 1946 (see [30,31]). The sample comprised 5,362 individuals (2,547 women) and data have been collected regularly since childhood. The representativeness of the study sample has been well documented [30,31]. A comparison of the sample retained at age 43 and 53 with population census data has shown that the NSHD survey members are generally representative of the national population of a similar age [32].

An annual sub-study of women's health in midlife was undertaken by postal questionnaire between the ages of 47–54. This study included 1,778 (70%) of the original cohort of women; the others had died (6%), previously refused to take part (12%) or lived abroad and were not in contact with the study or could not be traced (13%). The Ryff PWB was sent to the 1,421 women who had completed at least one women's health questionnaire in the previous 2 years. The representativeness of the sample of women who completed the Ryff items at age 52 has not been established in the same terms with respect to population census data. However, we compared the sample of women who completed the PWB and participated in the age 53 follow-up (N = 1108) or age 43 (where 53 data was not available (N = 57)) with those involved in the follow-ups but did not complete the PWB (n = 413). Ryff completers were of higher social class [chi-sq 16.6 df = 1, p < 0.001), more likely to be married (chi-sq 9.9 df = 1, p = 0.002) than non-completers and more educated (63.0 df = 1, p < 0.001). There was no difference due to employment status. This comparison excluded women (n = 50) who completed the Ryff items but neither the age 53 nor age 43 follow-ups. Comparative socio-demographic data was not available for the excluded group of women.

### Measures

#### Psychological well-being

A forty-two item version of the Ryff PWB was included in the women's health questionnaire at age 52 on the recommendation of C.Ryff (personal communication from C.Ryff to DK 1998). The response format for all items comprised six ordered categories labelled from 'disagree strongly' to 'agree strongly'. Twenty PWB items were positively worded and 22 negatively worded. Prior to analysis, negatively worded items were reverse scored so that high values indicated well-being. This made it easier to identify floor and ceiling effects. Full question wording of the 42-items is shown in Table 2.

**Table 2:** Response frequencies, Ryff 42-item Psychological Well-Being Scale (N = 1214*).

		1	2	3	4	5	6	Missing
**AUTONOMY**		%	%	%	%	%	%	%
A1+	I am not afraid to voice my opinions even when they are in opposition to the opinions of most people	4.0	11.5	10.7	19.9	**34.8**	16.7	2.7
A2+	My decisions are not usually influenced by what everyone else is doing	4.0	8.8	12.9	20.6	**34.9**	16.9	2.0
A4+	I have confidence in my opinions even if they are contrary to the general consensus	2.6	5.4	10.8	24.1	**35.8**	18.9	2.4
A6+	Being happy with myself is more important than having others approve of me	1.5	4.1	10.0	17.9	29.4	**35.2**	1.9
*A3-*	*I tend to worry what other people think of me*	*19.8*	*15.0*	*9.6*	***29.4***	*14.3*	*10.1*	*1.8*
*A5-*	*I often change my mind about decisions if my friends and family disagree*	*12.1*	*20.3*	*19.4*	***28.1***	*13.8*	*3.5*	*2.9*
*A7-*	*It is difficult for me to voice my own opinions on controversial matters*	***23.6***	*21.3*	*15.1*	*18.7*	*11.8*	*6.8*	*2.7*
**ENVIRONMENTAL MASTERY**								
E2+	I am quite good at managing the many responsibilities of my daily life	1.7	1.6	2.3	10.2	39.9	**42.5**	1.8
E4+	I generally do a good job of taking care of my personal finances and affairs	2.4	2.4	3.0	11.9	36.1	**42.0**	2.1
E5+	I am good at juggling my time so that I can fit everything in that needs to be done	2.9	6.0	7.0	14.9	**35.1**	32.0	2.1
E7+	I have been able to build a home and a lifestyle for myself that is much to my liking	2.7	2.5	5.6	16.3	33.5	**37.2**	2.1
*E1-*	*I do not fit very well with the people and the community around me*	***53.6***	*23.4*	*6.9*	*6.7*	*4.8*	*2.5*	*2.1*
*E3-*	*I often feel overwhelmed by my responsibilities*	***28.0***	*21.4*	*15.0*	*19.0*	*9.8*	*4.7*	*2.1*
*E6-*	*I have difficulty arranging my life in a way that is satisfying to me*	*25.2*	***25.5***	*15.7*	*16.6*	*10.0*	*4.9*	*2.3*

**PERSONAL GROWTH**								
G3+	I think it is important to have new experiences that challenge how you think about the world	2.2	4.3	8.4	24.3	**29.7**	**29.5**	1.6
G5+	I have the sense that I have developed a lot as a person over time	2.5	3.4	7.9	22.8	**34.4**	26.5	2.5
*G1-*	*I am not interested in activities that will expand my horizons*	***41.9***	*24.7*	*11.9*	*10.3*	*6.8*	*2.0*	*2.4*
*G2-*	*I don't want to try new ways of doing things – my life is fine the way it is*	*15.2*	***26.5***	*20.4*	*11.9*	*15.6*	*8.5*	*1.9*
*G4-*	*When I think about it, I haven't really improved much as a person over the years*	***33.3***	*27.5*	*13.2*	*9.6*	*10.0*	*4.5*	*1.8*
*G6-*	*I do not enjoy being in new situations that require me to change my old familiar ways of doing things*	*16.2*	*19.5*	*16.8*	***23.3***	*12.9*	*8.6*	*2.6*
*G7-*	*There is a truth in the saying that you can't teach an old dog new tricks*	***23.9***	*21.1*	*16.0*	*16.1*	*11.9*	*8.6*	*2.5*

**POSITIVE RELATIONS WITH OTHERS**								
R1+	Most people see me as loving and affectionate	1.6	2.5	5.3	15.1	**43.0**	29.9	2.6
R3+	I enjoy personal and mutual conversations with family members or friends	1.0	1.6	2.1	8.3	25.8	**59.7**	1.4
R6+	People would describe me as a giving person, willing to share my time with others	1.1	1.1	3.5	17.2	**37.2**	**37.6**	2.2
R7+	I know that I can trust my friends and they know that they can trust me	0.9	1.1	2.4	8.7	28.9	**55.8**	2.1
*R2-*	*I often feel lonely because I have few close friends with whom to share my concerns*	***42.8***	*18.6*	*9.6*	*13.5*	*7.1*	*7.1*	*1.3*
*R4-*	*I don't have many people who want to listen when I need to talk*	***36.4***	*25.0*	*11.2*	*12.2*	*9.3*	*4.1*	*1.7*
*R5-*	*It seems to me that most other people have more friends than I do*	***32.5***	*20.4*	*12.3*	*16.0*	*10.0*	*6.0*	*2.8*

**PURPOSE IN LIFE**								
P5+	I am an active person in carrying out the plans I set for myself	1.8	2.9	7.6	24.3	**35.0**	26.1	2.3
P7+	I enjoy making plans for the future and working to make them a reality	2.4	4.3	11.5	22.2	**30.6**	25.7	3.2
*P1-*	*I tend to focus on the present, because the future nearly always brings me problems*	***28.9***	*22.5*	*12.2*	*13.1*	*12.3*	*6.4*	*4.6*
*P2-*	*My daily activities often seem trivial and unimportant to me*	***34.7***	*23.0*	*10.5*	*15.3*	*9.4*	*5.2*	*2.0*
*P3-*	*I don't have a good sense of what it is I am trying to accomplish in life*	***27.9***	*22.7*	*11.8*	*17.4*	*11.5*	*5.5*	*3.1*
*P4-*	*I used to set goals for myself, but that now seems a waste of time*	***31.2***	*25.0*	*15.7*	*16.0*	*7.2*	*2.3*	*2.6*
*P6-*	*I sometime feel I have done all there is to do in life*	***50.7***	*20.9*	*10.8*	*7.6*	*4.4*	*3.5*	*2.1*

**SELF-ACCEPTANCE**								
S2+	I have made some mistakes in the past, but feel that all in all everything has worked out for the best	4.5	4.9	8.3	17.5	**33.0**	29.2	2.5
S5+	The past had its ups and downs, but in general I wouldn't want to change it	6.9	6.5	10.7	14.8	**30.3**	28.0	2.7
S6+	When I compare myself with friends and acquaintances, it makes me feel good about who I am	4.0	6.0	12.2	23.3	**30.3**	21.2	3.0
S7+	In general, I feel confident and positive about myself	5.4	6.8	10.0	14.1	**39.5**	21.0	3.2
*S1-*	*I feel that many of the people I know have got more out of life than I have*	***36.7***	*21.8*	*10.6*	*14.3*	*9.0*	*6.4*	*1.2*
*S3-*	*In many ways, I feel disappointed about my achievements in life*	***34.3***	*21.6*	*11.5*	*16.8*	*8.7*	*5.3*	*1.8*
*S4-*	*My attitude about myself is probably not as positive as most people feel about themselves*	*18.5*	*18.3*	*13.8*	***23.6***	*17.4*	*6.1*	*2.3*

Original coding, no recoding for positive and negative wording. 1. strongly disagree to 6. strongly agree.

*Italics = Questions with negative item content*. Modal response categories are highlighted in bold. Questions have been ordered by dimension, and do not reflect the order questions were asked to respondents. *Analysis Sample = 1,179 (includes subjects with data on at least 36/42 questions).

#### The General Health Questionnaire

One year after the Ryff items were completed, women survey members completed the 28 items of the "scaled" General Health Questionnaire [29]. The GHQ-28 is a multidimensional measure of psychological distress. The GHQ-28 comprises four sub-scales, Somatic symptoms, Anxiety/Insomnia symptoms, Social Dysfunction and Severe Depression, each with seven questions. Few of the items address positive aspects of function, although some items are positively worded [33,34]. A psychometric analysis conducted by the authors has shown that responses to GHQ-28 items in this cohort can be modelled in terms of four *a priori *first-order factors which all load (>0.80) on a higher (second) order latent factor capturing psychological distress.

### Psychometric modelling

#### Method of factor analysis

Confirmatory factor analyses were performed treating the six category PWB items as ordinal response variables. Model estimation was performed using robust Weighted Least Squares [26] (rWLS; estimator = Weighted Least Squares Mean and Variance adjusted (WLSMV)) procedures in M*plus *Version 3.13 [35]. Estimation using rWLS returns modified standard errors and a corrected chi-square test statistic of model fit. Unlike normal-theory maximum likelihood (ML) estimation for factor analysis of continuous scores, our use of Muthén's categorical data factor analysis methodology provides asymptotically unbiased, consistent and efficient parameter estimates, as well as a correct chi-square test of fit with dichotomous or ordinal observed variables [26]. To compare non-nested models, we report the sample size adjusted Bayesian Information Criteria (ssaBIC) from traditional linear factor analysis models that treat the ordinal responses as continuous (metric) variables (interval scores). In all models, individuals with partially missing item level data were included, since estimation of missing data patterns is possible under both estimators (traditional ML and WLSMV).

#### Stages in analysis

Models were estimated based on combinations of the following three model components: number of first-order factors (1 or 6); method factors (none, positive, negative, or both); second-order factors (present versus absent).

We introduced "method" factors in order to isolate nuisance variance due to item wording or content that was unrelated to the constructs being measured [15,16,36]. Inclusion of a method factor removed from the model any common tendency to respond similarly to PWB items with either positive or negative item content. Our method factors isolated between item-covariance orthogonal to the measured constructs. Technically these were assumed to be *uncorrelated *with the construct factors, and with each other. Each method factor was examined separately and then both were modelled simultaneously.

The magnitude of some inter-factor correlations reported by previous studies has given rise to the suggestion that the item-factor correspondences for some items are very weak; this can be tested by comparing the fit of the *a priori *measurement model, with one based on arbitrary allocation of items to factors (this is tantamount to saying that all measure well-being, but none measure any particular component or dimension of PWB). We generated four random item-factor models in order to evaluate the improvement of the *a priori *model over this scenario. We report the average fit statistics across the four solutions since all four random solutions were similar.

### Post-hoc modelling refinements

Further structural refinements were identified based on consideration of modification indices and a slightly revised model proposed (see results).

### Construct validity of the PWB constructs with respect to subsequent mental health

In order to examine the association between scores on the psychological well-being constructs, under our preferred model, and another measure of health (predictive validity), we linked the PWB scores for the women to their responses to the GHQ-28 conducted one year later.

## Results

Our analysis sample includes 1,179 respondents who completed at least 85% of PWB items (36 out of 42 questions); 957 had complete data on all items. Descriptive statistics revealed a general positive skew towards the well-being end of the response scales (Table 2). Responses to the most positive category were common (ranging from 12%–60%) and for just over half of the items this formed the modal category. These results indicated a ceiling effect on measurement of the individual items comprising the well-being scale. For questions including positive item content, responses to the lowest levels of well-being were few, often as little as 1–2% of responses to that question.

Each model is described in a single line in Table 3. This table includes a model reference number, the modelling components included, and fit statistics/information criteria.

**Table 3:** Model chi-square statistics (df) and goodness of fit criteria for Ryff 42-item Psychological Well-being Scale, N = 1,179

Model	1^st ^order factors	2^nd ^order	Method	Model	Chi Sq	Df	CFI	TLI	RMSEA	WRMR	SSABIC	SSABIC +/-
A0	6		No	Random	4,263.8	199	0.608	0.852	0.132	3.220	159,889	0.0
A1	1		No	Uni-dimensional	4,250.8	198	0.609	0.852	0.132	3.229	159,721	-168
A2	6		No	*A priori*	3,287.1	202	0.702	0.889	0.114	2.788	158,533	-1,356
A3	6	Yes	No	*A priori + 2^nd ^order*	3,332.7	202	0.698	0.888	0.115	2.846	158,621	-1,268
B1	1		P & N	Uni-dimensional	3,284.4	254	0.707	0.914	0.101	2.307	157,535	-2,354
B2	6		Negative	*A priori*	2,842.1	247	0.749	0.924	0.094	2.243	157,080	-2,809
B3	6		Positive	*A priori*	2,714.1	248	0.769	0.928	0.092	2.192	157,007	-2,882
B4	6		P & N	*A priori*	2.395.6	257	0.793	0.940	0.084	1.950	156,574	-3,315
B5	6	Yes	P & N	*A priori + 2^nd ^order*	2,460.6	255	0.787	0.937	0.086	2.010	156,467	-3.422

P&N – positive and negative method factors.

*CFI Comparative fit index (moderate to good fit >0.95) [41]*.

*TLI Tucker Lewis Index (moderate to good fit >0.95) [42]*.

*RMSEA Root Mean Square Error of Approximation (good fit <= 0.06) [43]*.

*WRMR Weighted root mean residual (good fit <1.0) [44]*.

*SsaBIC Sample size adjusted BIC statistic (lower numbers show improvement among non-nested models)*.

### Models A0-A3

Our first set of models (A0-A3, Table 3) tested the *a priori *model against a model with random item-factor associations (A0) and a unidimensional model with all 42 items loading on a single latent factor (A1). Here model A2 is the *a priori *model, and A3 is extended to incorporate a second-order factor (loaded by all six first-order factors).

Model fit was poor for all models in terms of all criteria (CFI <= 0.70; TLI < 0.90; RMSEA > 0.11; WRMR > 2.8) (Table 3). The worst model, in terms of the ssaBIC (highest value) was A0 with random factors. The *a priori *model (A2) returned a lower ssaBIC value than unidimensional model (A1). The model with a second-order factor (A3) returned a higher ssaBIC value than the *a priori *model with only first-order factors.

### Models B1-B5

Our second set of models (B1-B5) repeated A1-A3 with one, or both method factors. Compared to models A0-A3 any model incorporating either or both method factors improved model fit and substantially reduced the ssaBIC, regardless of the number of factors. Even in the model assuming a unidimensional construct of PWB (single first-order factor), but with both method factors, the ssaBIC dropped by a huge amount (>2000 points). In the *a priori *models with both method factors (B4, B5) RMSEA approached 0.08 and TLI approached 0.94, but CFI remained below 0.80. These two models (B4 *a priori *and B5 *a priori plus second-order*) were within 110 ssaBIC points but were indistinguishable on all other indices of fit.

### Interpretation of factor loadings from selected models

We report factor loadings from two models (A2 and B4) in columns 2 and 3 of Table 4. Inter-factor correlations for models A2 and B4 are shown as lower and upper diagonal entries in Table 5. In general, four factors were strongly associated (environmental mastery (E), personal growth (G), purpose in life (P), self-acceptance (S), but autonomy (A) and positive relations (R) were more distinct correlating <0.6 with these four constructs, and only 0.4 with each other. It is therefore particularly interesting to inspect the magnitude of the factor loadings for these four versus two constructs in the second-order model. In Table 6 we report the second-order factor loadings from these models; the two lowest loadings were for autonomy (A) and positive relations (R); all other loadings were 0.8 or above.

**Table 4:** CFA Model Estimates (M*plus *Estimator = WLSMV) for Ryff 42-item Psychological Well-being Scale, N = 1,179. a) Unstandardised Loadings (SE), b) Standardised Loadings

	**Model A2** *A priori *six-factor		**Model B4** Six-factor + two method factors		**Model PH2** Modified 40-item with two method factors	
	*a*	*b*	*a*	*b*	*A*	*b*
**Autonomy**						
A1 +	1.00 (0.00)	0.53	1.00 (0.00)	0.50	1.00 (0.00)	0.52
A2 +	0.98 (0.06)	0.52	0.92 (0.07)	0.46	0.94 (0.07)	0.49
A3 -	1.14 (0.07)	0.61	1.13 (0.09)	0.66	1.24 (0.09)	0.64
A4 +	1.29 (0.07)	0.69	1.16 (0.07)	0.58	1.16 (0.07)	0.60
A5 -	0.78 (0.06)	0.42	0.85 (0.07)	0.43	0.75 (0.07)	0.39
A6 +	0.92 (0.07)	0.49	0.76 (0.07)	0.38	0.72 (0.07)	0.40
A7 -	1.37 (0.07)	0.73	1.56 (0.10)	0.78	1.43 (0.09)	0.74
**Environmental mastery**						
E1 -	1.00 (0.00)	0.52	1.00 (0.00)	0.56	*Moved to R*	
E2 +	1.25 (0.07)	0.65	0.89 (0.07)	0.50	1.00 (0.00)	0.52
E3 -	1.06 (0.07)	0.55	1.06 (0.07)	0.59	1.12 (0.07)	0.58
E4 +	0.90 (0.07)	0.47	0.55 (0.07)	0.31	0.63 (0.06)	0.33
E5 +	1.01 (0.07)	0.55	0.64 (0.06)	0.36	0.72 (0.06)	0.37
E6 -	1.42 (0.08)	0.74	1.44 (0.08)	0.80	1.55 (0.09)	0.81
E7 +	1.44 (0.08)	0.75	1.18 (0.07)	0.66	1.33 (0.07)	0.69
**Personal growth**						
G1 -	1.00 (0.00)	0.49	1.00 (0.00)	0.45	1.00 (0.00)	0.36
G2 -	0.65 (0.06)	0.32	0.49 (0.07)	0.22	*Excluded*	
G3 +	1.04 (0.06)	0.51	1.02 (0.08)	0.46	*Excluded*	
G4 -	1.62 (0.09)	0.80	1.93 (0.14)	0.87	2.36 (0.23)	0.84
G5 +	1.70 (0.10)	0.83	1.52 (0.11)	0.69	1.97 (0.20)	0.71
G6 -	1.15 (0.07)	0.57	1.19 (0.09)	0.54	1.26 (0.12)	0.45
G7 -	0.80 (0.07)	0.40	0.75 (0.08)	0.34	0.65 (0.10)	0.23
**Positive relations**						
R1 +	1.00 (0.00)	0.46	1.00 (0.00)	0.30	1.00 (0.00)	0.32
R2 -	1.56 (0.11)	0.72	2.49 (0.27)	0.76	2.23 (0.23)	0.73
R3 +	1.38 (0.09)	0.63	1.65 (0.17)	0.50	1.57 (0.16)	0.50
R4 -	1.65 (0.10)	0.75	2.63 (0.27)	0.80	2.48 (0.24)	0.79
R5 -	1.64 (0.10)	0.75	2.64 (0.27)	0.80	2.51 (0.24)	0.80
R6 +	1.29 (0.08)	0.59	1.34 (0.14)	0.41	1.33 (0.13)	0.42
R7 +	1.31 (0.09)	0.60	1.58 (0.17)	0.48	1.55 (0.16)	0.49
E1 -					1.90 (0.20)	0.60
**Purpose in life**						
P1 -	1.00 (0.00)	0.43	1.00 (0.00)	0.45	1.00 (0.00)	0.41
P2 -	1.38 (0.09)	0.59	1.42 (0.10)	0.64	1.50 (0.11)	0.62
P3 -	1.53 (0.10)	0.66	1.58 (0.10)	0.71	1.69 (0.13)	0.70
P4 -	1.54 (0.10)	0.66	1.56 (0.10)	0.70	1.62 (0.12)	0.67
P5 +	1.61 (0.11)	0.69	1.19 (0.09)	0.53	1.32 (0.11)	0.55
P6 -	1.14 (0.09)	0.49	1.09 (0.09)	0.49	1.05 (0.10)	0.44
P7 +	1.52 (0.10)	0.65	1.23 (0.09)	0.55	1.36 (0.11)	0.56
**Self-acceptance**						
S1 -	1.00 (0.00)	0.76	1.00 (0.00)	0.80	1.00 (0.00)	0.78
S2 +	0.62 (0.03)	0.47	0.46 (0.03)	0.36	0.49 (0.04)	0.38
S3 -	1.05 (0.03)	0.80	1.07 (0.03)	0.85	1.09 (0.03)	0.85
S4 -	0.88 (0.03)	0.67	0.87 (0.03)	0.69	0.84 (0.03)	0.69
S5 +	0.79 (0.03)	0.60	0.64 (0.03)	0.51	0.68 (0.03)	0.53
S6 +	0.82 (0.03)	0.62	0.65 (0.03)	0.52	0.69 (0.03)	0.55
S7 +	0.96 (0.03)	0.73	0.83 (0.03)	0.66	0.86 (0.03)	0.67

+ positive item content, - negative item content

**Table 5:** Correlation Coefficients for Ryff 42-item Psychological Well-being Scale, N = 1,179

	***Upper diagonal = Model B4 - six-factor model with method factors***					
**Estimator = WLSMV**	**A**	**E**	**G**	**R**	**P**	**S**
A – Autonomy		***0.57***	***0.57***	***0.38***	***0.54***	***0.66***
E – Environmental mastery	0.62		***0.53***	***0.67***	***0.84***	***0.87***
G – Personal growth	0.62	0.61		***0.53***	***0.79***	***0.67***
R – Positive relations	0.42	0.70	0.60		***0.69***	***0.66***
P – Purpose in life	0.57	0.87	0.83	0.76		***0.86***
S – Self-acceptance	0.68	0.87	0.72	0.70	0.90	
	Lower diagonal = Model A2: six-factor model excluding method factor					
						
**Post Hoc Model (PH2)**	**A**	**E**	**G**	**R**	**P**	**S**
A – Autonomy		***0.57***	***0.55***	***0.38***	***0.50***	***0.64***
E – Environmental mastery			***0.63***	***0.62***	***0.82***	***0.85***
G – Personal growth				***0.65***	***0.82***	***0.78***
R – Positive relations					***0.74***	***0.69***
P – Purpose in life						***0.88***
S – Self-acceptance						
	***Upper diagonal = Model PH2: six-factor modified 40-item model with method factors***					

**Table 6:** Factor loadings from second-order model, Ryff 42-item Psychological Well-being Scale, N = 1,179.

		**A3**	**B5**	**PH3**
		**6F**	**6F + method**	**40 item**
**A**	Autonomy	0.67	0.63	0.61
**E**	Environmental mastery	0.90	0.88	0.87
**G**	Personal growth	0.79	0.80	0.83
**R**	Positive relations	0.75	0.74	0.74
**P**	Purpose in life	0.97	0.94	0.94
**S**	Self-acceptance	0.95	0.95	0.96

**A3**	Six-factor model (42-item)			
**B5**	Six-factor with method factors (42-item)			
**PH3**	Modified 40-item model with method factors			

### Post-hoc models

In a final round of modelling (Table 7) we found it useful to drop two items from personal growth (G) that exhibited a complex pattern of cross-loadings. Both of the excluded items, *G2 *(*I don't want to try new ways of doing things – my life is fine the way it is*) and *G3 *(*I think it is important to have new experiences that challenge how I think about myself and the world*) are complex questions, capturing more than one issue, and include both positive and negative item content. Item *E1 *(*I do not fit very well with the people and the community around me*) loaded more highly on positive relations (R) than its designated factor (environmental mastery (E)), reflecting the initial part of the question concerned with relationships with others. We therefore chose to model this item on positive relations (R).

**Table 7:** Post Hoc Models, Ryff Psychological Well-being Scale, modified models (40-item)

Model	Factors	2^nd ^order	Method	Model	Chi Sq	Df	CFI	TLI	RMSEA	WRMR	SSABIC	SSABIC +/-
PH1	6		No	Modified No method	2,635.1	197	0.753	0.915	0.102	2.477	150,403	0.0
PH2	6		P&N	Modified with method	1,678.5	252	0.856	0.961	0.069	1.613	148,661	-1.742
PH3	6	Yes	P&N	Modified 2^nd ^order	1,738.5	249	0.849	0.959	0.071	1.677	148,691	-1,711
PH4	6	Yes	P&N	Modified 2^nd ^order EGPS*	1,732.5	251	0.850	0.959	0.071	1.662	148,691	-1,712

P&N – positive and negative method factors.

*EGPS: Environmental mastery, personal growth, purpose in life, self-acceptance.

*CFI Comparative fit index (moderate to good fit >0.95) [41]*.

*TLI Tucker Lewis Index (moderate to good fit >0.95) [42]*.

*RMSEA Root Mean Square Error of Approximation (good fit <= 0.06) [43]*.

*WRMR Weighted root mean residual (good fit <1.0) [44]*.

*SsaBIC Sample size adjusted BIC statistic (lower numbers show improvement among nested models)*.

Examination of residuals also suggested potential overlap with two questions from positive relations (R) '*people would describe me as a giving person, willing to share my time for others*', and '*most people see me as loving and affectionate*' and so we allowed correlated residuals between these two items. These small modifications to the *a priori *model, together with method factors improved fit statistics for TLI and RMSEA (Models PH2 & PH3). The CFI however, still remained below 0.86.

**Figure 1:**
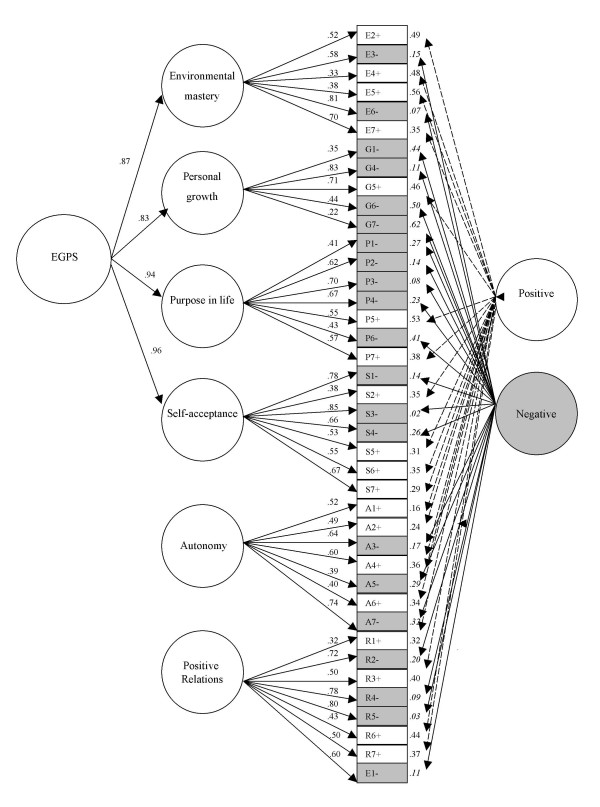
**Psychological well-being modified 40-item model, with second-order factor**. EGPS = general well-being factor comprising four first-order factors, environmental mastery, personal growth, purpose in life and self-acceptance. The model also includes residual correlation between R1 & R6 (not shown).

We also tested a six-factor model (PH4) where four constructs (environmental mastery (E), personal growth (G), purpose in life (P), self-acceptance (S)) loaded onto a second-order factor, and autonomy (A) and positive relations (R) remained as first-order factors (freely correlated). This model is drawn as a path diagram in Figure 1. Goodness of fit statistics for this model (PH4) were similar to the modified model (PH3) with all 6 constructs loading on the second-order factor. The distinctiveness of A and R from the four constructs that are most highly related (E,G,P,S) can be seen in the magnitude of the first-order factor inter-correlations from the modified model (PH2; Table 5) and the second-order factor loadings (PH3; Table 6) which were both less than 0.75 (50% common variance).

### Construct validity: predictive validity of the PWB for GHQ

The estimated correlation between our second-order PWB factor (model PH3 based on 40 items) and the GHQ-28 second-order factor was -0.45. The correlations among the *a priori *first-order PWB factors and the GHQ-28 second-order factor were low (-0.10–0.08) except for environmental mastery (E) (-0.52).

**Figure 2:**
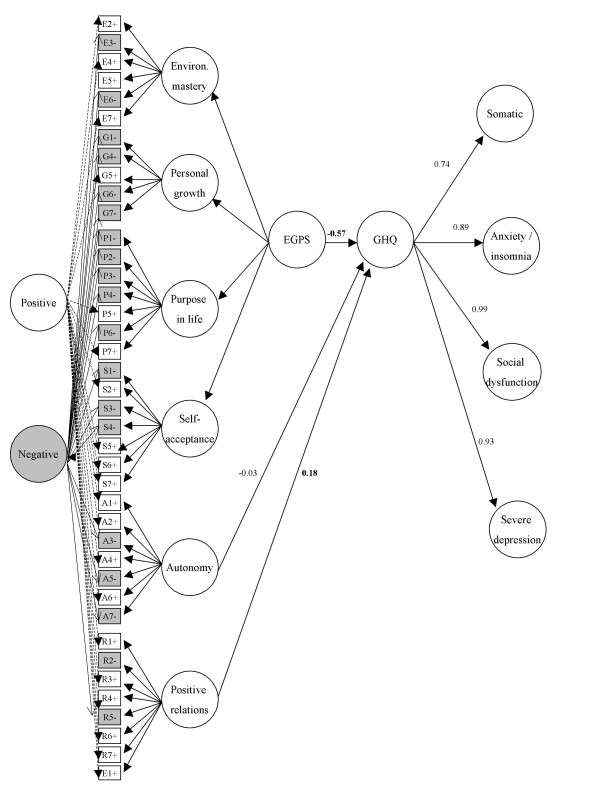
**External criterion validation of psychological well-being (modified 40-item model) with second-order GHQ-28**. Revised 40-item PWB model (PH4). EGPS = general well-being factor comprising four first-order factors, environmental mastery, personal growth, purpose in life and self-acceptance. Factor loadings for Ryff model are given in figure 1. The model includes residual correlation between R1 & R6. The GHQ first-order factors are comprised of 28 items (seven per sub-scale) (not shown). Correlations between Ryff six first-order constructs and second-order GHQ factor (*model not shown*): Autonomy -0.06, environmental mastery -0.52, personal growth -0.10, positive relations 0.08, purpose in life 0.08, self-acceptance 0.02.

Figure 2 shows that the correlation between the factors of our preferred Ryff model and second order GHQ (Model PH4) was -0.57 with the four first-order factors (E,G,P,S) loading on a second-order general well-being factor (model PH4).

## Discussion

In this study we provide the first confirmatory test of the factorial validity and structure of Ryff's Psychological Well-being (PWB) scales (42-item version) in the UK. In contrast to previous studies, our sample come from the UK and comprise only women who are surviving members of a national birth cohort study which began in 1946. This sample completed the Ryff items as part of an annual woman's health survey in midlife and also completed a mental health measure one year later.

In our psychometric modelling we evaluated the fit of categorical (ordinal response) factor models with single and six construct factors, first and second-order factors, and method factors, as well as providing a reference comparison to a model with random item-factor associations. Like all previous research we were unable to identify a model that fitted the data well, although a number of modelling components appeared to be useful in improving model fit to the data, and therefore determine our conclusions regarding the factorial validity of Ryff's measures, with reference to her theory, and in regard to these 42 items. Our results indicate the following:

1) We found conceptual and empirical value (improved model fit) from the addition of both positive and negative method factors to address methodological artefacts. Springer & Hauser [14] suggested the addition of a negative method factor (*correlated *with the construct factors) to the Ryff PWB model, but we extended this approach to the addition of both positive and negative method factors which were independent of both each other and the measured constructs. Models incorporating a single (either positive or negative) method factor offered an improvement over the same model without this feature, although models incorporating both methods factors had greater impact. Method factors introduce additional latent variables, and model parameters, but ssaBIC comparisons show that these modelling additions improve fit despite penalties for the improvement in the log-likelihood value achieved by the estimation of additional parameters. However we note that these BIC values are taken from traditional linear factor models (since BIC values are not available for WLS solutions).

2) Regarding dimensionality of the PWB measure, and empirical associations among the *a priori *constructs, we found that in our sample four of the six dimensions of well-being (environmental mastery (E), personal growth (G), purpose in life (P), and self-acceptance (S)), as operationalised by these 42 items, were sufficiently highly correlated to warrant introduction of a general well-being factor, as a second-order general factor, that explained the association among the first-order constructs. We could not justify the inclusion of the remaining two dimensions (autonomy (A) and positive relations (R)) on this second-order construct since they were more independent of these four factors, of the second-order factor, and of each other. This gives some credence to claims that there are fewer than six dimensions under-pinning Ryffs PWB items. However our interpretation is in terms of the hierarchical organisation of the six factors, which seem to span two conceptual levels [7]. Further replications of this structure are warranted.

3) Finally, we found a strong negative association between a measure of mental health (severity of psychological distress based on the GHQ-28) and the PWB which were measured one year apart. The major contribution to this predictive association came from the environmental mastery items. This replicates a finding reported by Ryff & Keyes [7] using cross-sectional data. A possible explanation of this finding from attribution theory is that people who perceive their environment as uncontrollable, i.e. score low on the environmental mastery construct, and attribute this lack of control to some internal cause that is global and stable, feel helpless to prevent future negative outcomes and consequently experience depression [37,38]. There is also some overlap in item content to do with task-related and role functioning between Ryff's environmental mastery items and some items in the GHQ-28. Validation against more objective measures could be useful, since most data concern other self-report questions

We tested the fit of random-item factor models in our data. Our random item-factor models differed by 1,356 BIC points from the ssaBIC for the theoretical model. This indicates to us that there are still some fragile item factor associations in the six-factor model, otherwise this comparison would yield a much larger reduction in BIC when comparing theoretical to random models.

Previous authors have concluded that the empirical data are not consistent with a six-factor model [11]. We do not reject the six construct factors, but see the value of a more parsimonious model, based on a hierarchical representation of the proposed dimensions. This approach is common in mental health epidemiology and personality research but does not seem to be as frequently adopted in well-being literature.

Our second-order factor model requires the item to factor mapping established for the first-order factors, for its definition, since it is the second-order (more general) factor that is proposed as the explanation for the association among environmental mastery (E), personal growth (G), purpose in life (P), and self-acceptance (S). Examination of item content suggests that this second-order factor may encapsulate a motivational aspect of well-being which incorporates notions of goal orientation and self-direction. Our finding that there are three (rather than six) distinct factors – autonomy, positive relations and motivation/self-direction – is reminiscent of the work of Deci and Ryan [39,40] which postulates that well-being results from the fulfillment of three basic psychological needs – autonomy, relatedness and competence. It could be argued that our second-order factor bears a relationship to Deci and Ryan's concept of competence. However it should be noted that while there is overlap between the autonomy concepts of Ryff and of Deci & Ryan, the latter focus on the core concept of personal control while Ryff's items include an element of not caring what others think. The three factor structure of well-being has also suggested by Kafka & Kozma [11]. Their factor analyses of Ryff PWB (120-items) (See table 1 for details) which also included the Satisfaction with Life Scales (SWLS) and Memorial University of Newfoundland Scale of Happiness (MUNSH) extracted main three factors, with the first comprising environmental mastery (E), personal growth (G), purpose in life (P), and self-acceptance (S); the second MUNSH & SWLS and the third factor autonomy (A) and positive relations (R). Given these results we advocate a more parsimonious approach to examine antecedents and correlates of general well-being, as defined by the second-order factor, and/or to examine the specific antecedents or correlates of the first-order factors. These are scientific questions at different levels of generality, and should be recognized as such.

Many aspects of our modelling results do suggest that some Ryff items may measure more than one of the six constructs in the theory. This possibility requires further theoretical work that was not undertaken here, but should form an agenda for future research, and for future factorial complexity studies.

### What are the implications of our factor analysis results for users of the Ryff PWB scales?

The factor loadings from our preferred model (Figure 1) indicate that many item-factor loadings for Ryff PWB items on the six construct factors are generally low, with only 11/40 exceeding 0.70. This would suggest that shortening this version of the PWB may not be practically possible, since the item reliabilities for almost three-quarters of the items are probably too low to allow for reliable estimation of construct scores. We note that others have suggested shorter versions, or motivated the need for them to reduce respondent burden in well-being surveys [12]. Existing short versions, e.g. the 18-item PWB do not include many items from the 42 analysed here.

Related to this observation, Figure 1 shows that 3 out of the 6 first-order factors have only 1 high factor loading (>0.70) indicating that the underlying construct explains only 50% of the variance in item response. This brings into question the definition of the constructs in terms of these single high loading items. These results suggest to us that future studies should continue to examine internal construct validity of the PWB items. They also indicate that the items in this version, and perhaps other long versions, are not sufficient to define robust latent constructs: more items with high loadings should increase the stability of the factor solutions recovered across different study samples (*we thank an anonymous referee for distinguishing these two suggestions*).

Applied researchers who do not wish to execute complex latent variable models will not be able to distinguish contributions to variance from method versus construct sources and are at a disadvantage in terms of their ability to define and refine both conceptually relevant and psychometrically important variants of multidimensional scale analysis. However, in these instances, a parsimonious account of associations of other variables with Ryff PWB outcomes may be achieved by adding all items loading on the four factors that define our second-order well-being continuum (EGPS; Environmental mastery, personal growth, purpose in life and self-acceptance). In samples similar in composition to ours, researchers might wish to consider using our factor loadings as weights, to form sum scores using our loadings in Figure 1. Further insights into the latent structure of the Ryff items will require equally complex models and replications of these results with method factors. In other areas of multivariate statistics the role of model-based analyses is also central e.g. missing data modelling using maximum likelihood.

The nature of our data ensures we are undertaking a pure test of the structure of PWB items since our sample are homogeneous with respect to age and gender (women age 52). Our study design therefore minimises the impact of socio-demographic characteristics. Although our sample suggested Ryff completers were more likely to be of higher socio-economic backgrounds than non-completers, comparative studies using the PWB in nationally representative samples e.g. MIDUS and NSFH [7,14] do not report details regarding representativeness or non-completion. Therefore, it is not possible to assess whether the imbalance noted in our sample is likely to be present in other nationally representative samples of PWB using self-completion methods. Further, it could be argued that our conclusions regarding the latent structure of Ryff PWB items may be unique to this cohort and to the 42-item version of the Ryff PWB, but we believe that our results are similar enough to other studies to suggest that our psychometric conclusions and modelling innovations have validity outside of this sample.

Future research could apply further psychometric refinement to the Ryff PWB dimensions, by exploring scoring and effective measurement range using item response theory methodology.

## Conclusion

Our psychometric analyses of the Ryff 42-item PWB suggests that the addition of two method factors to reflect positive and negative item content improves model fit. A revised model with a single second-order factor, loaded by four of the six first-order factors (environmental mastery, personal growth, purpose in life and self-acceptance), two method factors, and two more distinct first-order factors (autonomy and positive relations) provided the most parsimonious solution in this birth cohort sample. Psychological well-being was negatively associated with mental health, but further investigation of precision of measurement across the health continuum is required.

## Abbreviations

SWB subjective well-being

PWB psychological well-being

A autonomy

E environmental mastery

G personal growth

R positive relations with others

P purpose in life

S self-acceptance

NSHD National Survey of Health and Development

GHQ General Health Questionnaire

RMSEA Root Mean Square Error of Approximation

TLI Tucker Lewis Index

CFI Comparative Fit Index

WLSMV Weighted Least Squares Mean Variance adjusted

RWLS Robust weighted least squares ssaBIC Sample size adjusted Bayesian information criteria

ML Maximum likelihood

## Competing interests

The author(s) declare that they have no competing interests.

## Authors' contributions

RAA undertook the statistical modelling, manuscript preparation and revision. TJC planned and advised on the statistical modelling using latent variables and participated with manuscript preparation and revision. GBP was actively involved with the statistical modelling; FAH advised on the conceptual framework and data interpretation and DK and MEW were responsible for the design and acquisition of the data. FAH, GBP, DK, MEW all advised on manuscript preparation.
